# Predictive value of foetal superior temporal sulcus asymmetry for neonatal speech discrimination

**DOI:** 10.1093/braincomms/fcag048

**Published:** 2026-02-13

**Authors:** Sophie Mandl, Patric Kienast, Gregor Kasprian, Florian Ph S Fischmeister, Anna Weiskopf, Estella Ringelmann, Johannes Tischer, Michael Weber, Apeksha Hadole, Rainer Seidl, Lisa Bartha-Doering

**Affiliations:** Department of Pediatrics and Adolescent Medicine, Comprehensive Center for Pediatrics, Medical University of Vienna, Vienna 1090, Austria; Division of Neuroradiology and Musculoskeletal Radiology, Department of Biomedical Imaging and Image-Guided Therapy, Medical University of Vienna, Vienna 1090, Austria; Division of Neuroradiology and Musculoskeletal Radiology, Department of Biomedical Imaging and Image-Guided Therapy, Medical University of Vienna, Vienna 1090, Austria; Division of Neuroradiology and Musculoskeletal Radiology, Department of Biomedical Imaging and Image-Guided Therapy, Medical University of Vienna, Vienna 1090, Austria; Department of Pediatrics and Adolescent Medicine, Comprehensive Center for Pediatrics, Medical University of Vienna, Vienna 1090, Austria; Department of Pediatrics and Adolescent Medicine, Comprehensive Center for Pediatrics, Medical University of Vienna, Vienna 1090, Austria; Computational Imaging Research Lab, Department of Biomedical Imaging and Image-Guided Therapy, Medical University of Vienna, Vienna 1090, Austria; Division of Neuroradiology and Musculoskeletal Radiology, Department of Biomedical Imaging and Image-Guided Therapy, Medical University of Vienna, Vienna 1090, Austria; Division of Neuroradiology and Musculoskeletal Radiology, Department of Biomedical Imaging and Image-Guided Therapy, Medical University of Vienna, Vienna 1090, Austria; Department of Pediatrics and Adolescent Medicine, Comprehensive Center for Pediatrics, Medical University of Vienna, Vienna 1090, Austria; Department of Pediatrics and Adolescent Medicine, Comprehensive Center for Pediatrics, Medical University of Vienna, Vienna 1090, Austria

**Keywords:** foetal MRI, functional near-infrared spectroscopy, language development, neonates, speech discrimination

## Abstract

While some anatomical brain asymmetries are seen across primates, the earlier appearance and larger depth of the right superior temporal sulcus (STS) is specific to human foetuses. Interestingly, the degree of STS asymmetry varies between foetuses, and it has been shown that this interindividual variability is related to the functional lateralization of the language network in school-aged children. It remains unclear, however, whether it is also indicative of language localization and functioning shortly after birth. In the present longitudinal study, we prospectively examined the predictive value of foetal STS asymmetry for neonates’ language lateralization and neural speech discrimination. We measured the STS depths and volumes in neurotypical foetuses (*N* = 35) using foetal MRI. After birth, we investigated the neonates’ haemodynamic response to forward and backward speech using functional near-infrared spectroscopy. We hypothesized that less rightward asymmetry of the STS depths in the foetal brain is related to increased left language lateralization and to a greater haemodynamic difference between speech conditions in the left hemisphere in neonates. While the foetuses demonstrated an overall rightward asymmetry of the STS depths and volumes, the degree of asymmetry varied between individuals. After birth, the group activated left frontal and right temporal regions during the speech discrimination paradigm. Again, there was variability in the degree of neural activation in response to speech. Importantly, we found that children with a foetal STS depth asymmetry towards the left hemisphere activated their right hemisphere less for forward speech (*r* = −0.58, *P* = 0.002) and differentiated less between forward and backward speech in their right hemisphere (*r* = −0.48, *P* = 0.014). Contrary to our initial hypothesis, the results suggest that an earlier structural development of the left temporal lobe goes along with reduced involvement of the right hemisphere, rather than an increased involvement of the left hemisphere, during neural speech discrimination. Given that rightward asymmetry of language-related brain areas has been associated with weaker language abilities, the present study provides important preliminary data regarding the neural underpinnings of language development during the prenatal and early postnatal period.

## Teaser

Mandl *et al*. report that less rightward asymmetry of the foetal superior temporal sulcus depths is associated with reduced activation in the right hemisphere during neonatal speech discrimination. Given that rightward asymmetry of language-related brain areas has been associated with weaker language abilities, this study provides important information regarding the neural underpinnings of early language development.

## Graphical Abstract

**Graphical Abstract:**
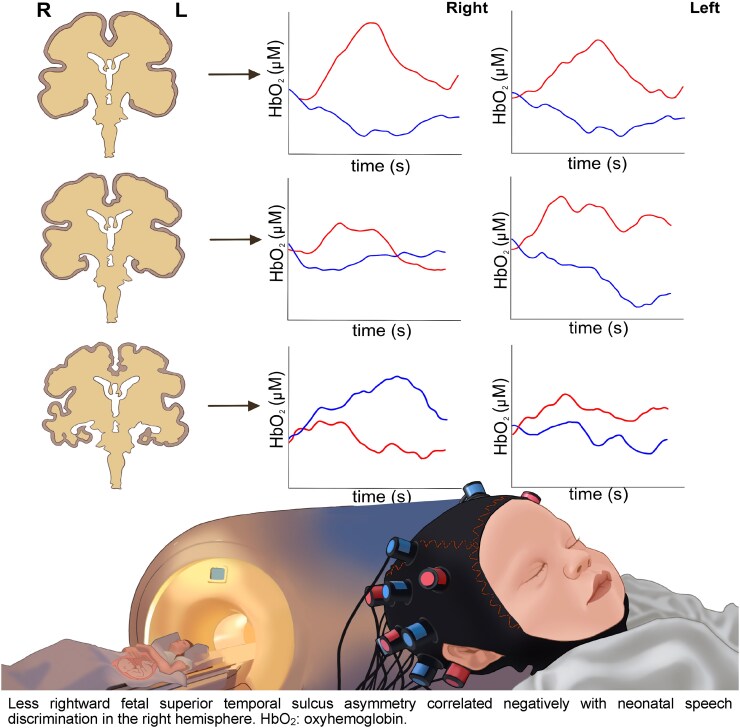

## Introduction

Starting around the 23rd week of pregnancy, a left–right hemispheric asymmetry of perisylvian regions becomes apparent in the foetal brain.^1^ Some of these anatomical asymmetries are seen in non-human primates as well, including a longer Sylvian fissure, and a larger planum temporale and arcuate fasciculus in the left hemisphere.^2^ The earlier appearance and larger depth of the right superior temporal sulcus (STS), however, is specific to the human brain.^3,4^ Post-mortem and *in vivo* foetal MRI studies have repeatedly demonstrated that the right STS develops 1–2 weeks earlier than its left counterpart and is significantly deeper in 94% of human foetuses.^5-7^ In healthy brains, these anatomical asymmetries sustain throughout infancy and remain relatively stable until adulthood, yet they vary interindividually.^8,9^ Moreover, they are assumed to be linked to the lateralization of cognitive functions in the brain.

Aside from the motor system, language is the most lateralized function in the human brain, with left lateralization already becoming apparent in early infancy, particularly during phonetic-phonological discrimination tasks. Studies in healthy, full-term neonates employing functional near-infrared spectroscopy (fNIRS) and a forward–backward speech paradigm have revealed a bilateral language network that encompasses frontal and temporal brain regions, with a stronger haemodynamic response to forward speech than backward speech in the left hemisphere.^10-12^ Functional MRI studies in 2- to 3-month-old sleeping infants have similarly reported greater activation by forward than backward speech and by speech than music in the left planum temporale than in its right counterpart.^13,14^

It is important to note that, while language is strongly lateralized to the left hemisphere in adults, this functional organization is still developing in the infant brain, which means that the degree to which infants lateralize can vary and lead to mixed study results. For example, a recent fMRI study found a slightly right-lateralized activation pattern for forward versus backward speech in the overall group of infants.^15^ However, the same study also found that children who already displayed stronger left lateralization in infancy exhibited better language skills 5 years later. In fact, increased left language lateralization has repeatedly been associated with better language functioning.^16-18^ Atypical functional symmetry or functional asymmetry of language-related brain regions towards the right hemisphere, on the other hand, has been implicated in neurodevelopmental disorders that are accompanied by language deficits, including specific language impairment,^19^ dyslexia^20^ and autism spectrum disorder.^21^

Similarly, atypical anatomical asymmetry is assumed to be a risk factor for neurobehavioral disorders.^22,23^ For example, abnormal temporal structural asymmetries have been associated with neurological and psychiatric diseases such as autism spectrum disorder,^21^ schizophrenia^24^ and dyslexia.^25^ The extent to which anatomical asymmetries found in the foetal temporal lobe are predictive of language development, however, is less known. Given the human-specific anatomical asymmetry of the STS, STS asymmetry has been put forth as a potential anatomical marker for language abilities. The STS separates the superior temporal gyrus (STG) from the middle temporal gyrus in the temporal lobe, and it has been implicated in language processing in the left hemisphere and social cognition in the right hemisphere.^26,27^ More specifically, the left STS is involved in linguistic functions such as phonological processing, whereas the right STS plays a role in voice recognition, gaze perception, and theory of mind.^27-29^ Only one study has examined the association between foetal STS asymmetry and later language abilities longitudinally.^30^ This study found that healthy foetuses with less rightward asymmetry of the STS depths had better verbal abilities and increased left language localization 6–13 years later.

Offering early intervention services can alter the developmental course. It is therefore of interest to examine language functioning as early as possible. Since the neural language network can already be studied in newborns, it may be posited that foetal STS asymmetry is also predictive of very early language lateralization and functioning. To the best of our knowledge, no study so far has prospectively examined this relationship. Therefore, the present study aimed to investigate whether the asymmetry of the foetal STS depths predicts neonates’ language lateralization and neural speech discrimination using fNIRS and a forward–backward speech paradigm. We hypothesized that foetal STS depth asymmetry towards the left hemisphere is related to increased left language lateralization and to a greater haemodynamic difference between speech conditions in the left hemisphere in neonates.

## Materials and methods

### Participants

Thirty-five participants whose mothers had undergone foetal MRI diagnostics for clinical reasons participated in the study between 2022 and 2024. Inclusion criteria were (i) a foetal MRI examination between 22 and 35 weeks of gestation, and (ii) native German speaking parents. Exclusion criteria were (i) general MRI contraindications (i.e. implants, cardiac pacemakers), (ii) claustrophobia, and (iii) neurological findings. The study was approved by the Ethics Committee of the Medical University of Vienna and performed in accordance with the Helsinki Declaration of 1975. Mothers gave informed written consent before their child’s inclusion.

### Foetal MRI acquisition

Foetal MRI examinations were performed at the Department of Radiology, Division of Neuroradiology, Medical University of Vienna on a Philips Ingenia 1.5 T scanner according to the international guidelines of foetal MRI.^31^ Multiple structural T_2_-weighted sequences (variable repetition time: 14 000–25 000 ms, echo time: 100–140 ms, slice thickness: 3–4.4 mm, field of view: 200–230 mm, matrix: 256 × 256, in-plane resolution: 0.72/0.72/4.00 mm, flip angle: 90°, acquisition time: 15–19 s) were acquired in three orthogonal planes. All foetal scans were acquired between the 23rd and the 36th week of gestation (Supplementary Fig. 1). Two neuroradiologists (G.K. and P.K.) performed an image quality check prior to study inclusion.^32^ Therefore, all 35 cases included in the current study were considered to have good or excellent image quality.

### Foetal STS depth measurements

The coronal T_2_-weighted sequence with the highest quality was used to measure the maximum depth of the foetal STS in each hemisphere as first described in Kasprian *et al.*^5^ (Supplementary Fig. 2). The quality of the coronal sequence was based on orthogonality, visibility of the inner ear structures, and motion artefacts. Therefore, we measured the STS depths in the coronal sequence with exact orthogonal orientation, symmetric position of the cochleas, and no motion artefacts. Three raters (S.M., P.K., and G.K.) independently determined the sulcal depth of both STS by first drawing a line conjoining the vertex of the developing STG and the developing middle temporal gyrus and then measuring the distance between the deepest point of the STS and the drawn line. To determine the inter-rater reliability of the STS depth measurements, an intraclass correlation coefficient (ICC) was calculated using a two-way mixed-effects model with absolute agreement. Excellent agreement was observed among the three raters, with an average-measure ICC of 0.986 [95% CI: 0.973–0.993; *F*(24,48) = 69.203, *P* < 0.001]. Given this high reliability, the three ratings for each foetal STS depth were averaged, and the asymmetry index (AI) was determined using the formula: AI = [((Left − Right)/(Left + Right)) × 100]. An AI value of +100 corresponds to complete left- and an AI value of −100 corresponds to complete right hemisphere dominance of STS depth. AI was categorized as left lateralized if ≥20, bilateral if within −20 and +20, or right if ≤−20.

### Super-resolution reconstruction and foetal brain volumetry

Multiple anisotropic image stacks from each foetal brain were combined into single 1 mm isotropic volumes using the neural slice-to-volume reconstruction (NeSVoR; Version 0.5.0) algorithm.^33^ First, the NeSVoR pipeline preprocessed the selected T_2_-weighted sequences via automatic brain masking and bias field correction, and conducted an image quality assessment of the input stacks. The image quality assessment examines artefacts, such as motion, and rates each image stack from 0 to 1, where a score of 1 indicates a motion-free image. The five best image stacks were then combined using slice-wise motion correction and volumetric super-resolution reconstruction.

The resulting super-resolution reconstructions were automatically segmented using the Brain vOlumetry and aUtomated parcellatioN for 3D feTal MRI (BOUNTI) pipeline.^34^ The BOUNTI pipeline subdivides the brain into 19 regions of interest, including cerebrospinal fluid, cortical grey matter, white matter, deep grey matter, ventricles, cavum, brainstem, and cerebellum. All automatic segmentations were checked and manually corrected using the segmentation tool in ITK-Snap (Version 3.8.0). The STS was then manually segmented from the most anterior point of the STS to the posterior point where the STS curves upwards towards the angular gyrus by measuring the cortical areas bordering the STS in each hemisphere.^35^ To quantify structural brain asymmetry, the cortical grey matter, white matter, deep gray matter, and STS volumes were calculated in each hemisphere. The AI of each anatomical region was then calculated as described in the previous section.

### Speech discrimination paradigm

We administered an adapted version of the paradigm first described in Peña *et al.*,^10^ which investigates neural speech discrimination in neonates by comparing forward and backward speech and has already shown robust findings in neonates.^11,36-38^ A female speaker was recorded while she recited the German children’s story ‘Little-I-Am-Me’ using infant-directed speech. The recording was then edited to 10 sequences of 15 s with well-formed and complete prosodic units each (mean pitch = 233 Hz). The mean intensity of the sequences was equalized (mean intensity = 70 dB). Each sequence was time-reversed using version 2.1.1. of Audacity^®^ recording and editing software (Audacity, 2012). This generated 10 additional backward speech stimuli with the same acoustic and phonetic features but with distorted phonological, semantic, and prosodic information, resulting in 20 sequences total. Each sequence was followed by a silence with randomized length between 25 and 30 s and the order of sequences was pseudo-randomized with no more than two consecutive sequences from the same condition. The overall duration of the speech discrimination paradigm was 14 min and 40 s.

### Neonatal fNIRS data acquisition and processing

A continuous wave fNIRS system (NIRSport2, NIRx, Medical Technologies) with LED optodes (760 and 850 nm wavelengths) was used to measure cortical activity at a sampling rate of 10.2 Hz. The optical probes contained 10 sources and 8 detectors, which were inserted into an elastic cap (Easycap, Brain Products GmBH, Germany) and then placed on the neonates’ head using the vertex, tragus, and ears as surface landmarks. The resulting 2 × 12-channel configuration covered bilateral frontal, temporal, and parietal regions, with a mean distance of 3 cm between each source–detector pair.

Before the experiment, all neonates were bottle- or breastfed by their mothers to ensure that they were sleeping or resting with their eyes closed. Testing took place in a quiet, dimly lit room. The stimuli were presented using two loudspeakers positioned at a distance of ∼2 m in front of the neonate and at a 30° angle from the neonate’s head. Acquisition took place after birth, between the 37th and the 45th week of gestation.

The fNIRS data were processed using the open-source software HomER3, which is implemented in MATLAB (R2019b, MathWorks, Natick, MA, USA). First, channels with low signal-to-noise ratio (<2) were removed from individual participants’ datasets. Study participants with <16 out of 24 (66.7%) channels remaining post-processing were excluded from further analyses. The final analyses thus included 25 infants. The raw optical intensity data series was then converted into changes in optical density data. Motion artefacts were automatically identified in each channel and corrected using both the Spline and wavelet motion correction algorithms, as recommended by Di Lorenzo *et al*.^39^ To eliminate high-frequency instrument noise, the optical density data were low-pass filtered with a cut-off frequency of 0.5 Hz. Baseline drifts were eliminated using a high-pass filter of 0.01 Hz. Changes in the concentration of oxyhaemoglobin (HbO_2_) and deoxyhaemoglobin (Hb) were calculated from changes in optical density using the modified Beer–Lambert law and neonate-specific differential pathlength factor (DPF) values: DPF(760) = 5.29, DPF(850) = 4.23.^40^ The haemodynamic response function was extracted for each subject, channel, and condition (forward/backward speech) by calculating the mean response from 2 s before stimulus onset to 20 s after stimulus onset. Changes in HbO_2_ and Hb were then averaged over a 15-s time window starting from stimulus onset and exported for subsequent analyses. This time window was selected based on visual inspection of the current data to include the range of maximum HbO_2_ concentration changes observed across the neonates. Since HbO_2_ is supposed to be more sensitive to blood oxygenation changes associated with neonatal brain activity than Hb, further analyses were conducted using HbO_2_.^41,42^ All Hb results can be found in the Supplementary Note 1.

To determine which regions significantly activated in response to speech, a non-parametric cluster-based permutation test was performed.^43^ First, one-sample *t*-tests for forward speech and for backward speech were conducted against the zero baseline for each channel and each timepoint (sampling rate = 10.2 Hz). The *t*-score threshold was set at *t* = ±2. All samples within a channel whose *t*-score exceeded this threshold were selected and clustered based on temporal adjacency. For each channel, the *t*-values within each temporal cluster were summed and the cluster with the maximum *t*-value was taken. Within each channel, the pair of the forward/backward speech condition and the zero baseline was then randomly relabelled. For each permutation, the same procedure was carried out as for the originally labelled data. This procedure was repeated 2000 times to generate a probability curve of *t*-values for each channel. The original *t*-value was then compared with this H_0_ distribution to test whether the null hypothesis is true (i.e., forward/backward speech does not result in a significantly different haemodynamic response from the zero baseline in the given channel). To calculate cluster-level statistics, we identified all possible sets of four quadrilateral nearest-neighbouring (≤3 cm) channels along the fNIRS array.^44^ These clusters were quantified by summing the multiple time sample-specific *t*-values of each channel within each cluster candidate. A new H_0_ distribution for each cluster candidate was then generated, and the multiple sample-specific *t*-value of each cluster was compared with this H_0_ distribution to see whether it was significantly different from chance. Within each hemisphere, the cluster with the smallest significant *P*-value was selected as the primary region of interest for each speech condition.

### Statistical analyses

All statistical analyses were performed using IBM SPSS Statistics (Version 29.0). Mean HbO_2_ and Hb concentrations were normally distributed, whereas the STS depths, volumes, and AI did not follow a normal distribution. However, since the residuals of the STS depths, volumes, and AI were normally distributed, we performed parametric statistical tests. For the fNIRS group analyses, paired *t*-tests were used to compare neonates’ haemodynamic responses to forward and backward speech across all channels in each hemisphere and within significant clusters. To evaluate if neural speech discrimination between conditions within each hemisphere was significant on an individual level, we also performed paired *t*-tests separately for each subject. We further calculated an individual lateralization index (LI) of neural activity during speech discrimination in the overall brain by using the formula: LI = [((|Left| − |Right|)/(|Left| + |Right|)) × 100], with −100 representing complete right-hemispheric dominance and +100 representing complete left-hemispheric dominance of neural speech discrimination. Since oxyhaemoglobin signals represent relative changes from baseline and can take negative values, the absolute difference between conditions was used for the LI formula.^36^

To test our hypothesis regarding the relationship between foetal structural brain asymmetry and speech discrimination at birth, confirmatory analyses included calculating the Pearson correlation coefficient between the AI of the STS depths and (i) the functional lateralization of neural speech discrimination, as well as (ii) neural speech discrimination within each hemisphere. As the values of neural speech discrimination within each hemisphere define the functional lateralization of speech discrimination and are therefore statistically dependent, we did not apply a correction for multiple comparisons in the confirmatory analysis.

In addition, exploratory analyses using Pearson’s correlation were conducted to examine whether foetal STS depth asymmetry was also associated with the infants’ haemodynamic response to the individual speech conditions (forward/backward), and whether foetal STS volume asymmetry correlated with neural speech discrimination, forward speech, or backward speech. To control for multiple comparisons in the exploratory analysis, *P*-values were adjusting using the Bonferroni correction (i.e. *α* = 0.05/4 = 0.0125).

Gestational age at the time of the foetal MRI examination and at the time of the fNIRS measurement did not significantly correlate with the AI of the STS depths or volumes, nor with the mean HbO_2_ concentrations (all *P* > 0.050). However, to rule out any possibility that age at scan might influence the results, partial correlation coefficients were calculated while controlling for gestational age at scan (*r*_p_*)*.

## Results

Overall, 35 pregnant women with neurologically healthy foetuses were prospectively recruited for this study at the time of their foetal MRI examination. Their newborns were then tested with fNIRS and the speech discrimination paradigm within the first 4 weeks after birth. Ten neonates were excluded due to a poor signal-to-noise ratio in more than eight channels (>33.3%) during fNIRS acquisition. Clinical data of the remaining 25 neonates (13 female/12 male) are depicted in Table 1.

**Table 1:** Participant data (*N* = 25)

	Mean (SD)/Median^a^	Range
**At foetal MRI examination**		
Gestational age (weeks)	27.49 (3.54)	22.57–35.43
Right STS depth (mm)	2.46 (2.31)	0.00–8.40
Left STS depth (mm)	1.52 (2.16)	0.00–7.80
Asymmetry index of the STS depths	−43.35 (42.49)	−100.00–13.46
Right STS volume (cm^3^)	0.32 (0.31)	0.00–0.59
Left STS volume (cm^3^)	0.14 (0.21)	0.00–1.27
Asymmetry index of the STS volumes	−48.59 (42.40)	−100.00–0.00
**At birth**		
Gestational age (weeks)	39.08 (2.02)	34.00–41.29
Head circumference (cm)	33.88 (1.60)	30.00–37.00
Weight (g)	3122.44 (629.72)	1938.00–4010.00
Length (cm)	50.03 (3.68)	42.00–55.00
Apgar score at 10 min	10	7–10
**At fNIRS assessment**		
Gestational age (weeks)	41.59 (2.18)	36.86–44.86
Head circumference (cm)	35.07 (1.41)	33.0–37.0
Weight (g)	3371.36 (581.40)	2500.00–5056.00
Length (cm)	51.63 (2.73)	47.00–57.00

^a^The median was only calculated for the Apgar score. All other values depict the group mean.

### Structural asymmetry in the foetal brain

Whereas cortical grey matter, white matter, and deep grey matter volumes did not significantly differ between hemispheres, the volumes and depths of the STS showed significant hemispheric asymmetry (Table 1; for more details, see Supplementary Tables 1 and 2).

On the group level (*N* = 25), the mean right STS was significantly deeper than the mean left STS [*t*(24) = −5.49, *P* < 0.001], with the AI showing an overall right asymmetry of the STS depths. On the subject level, the AI of the STS depths ranged from −100.00 to +8.11, with 16 foetuses being right-lateralized and 9 foetuses showing symmetrical STS depths. Similarly, the group mean volume of the right STS was significantly larger than the group mean volume of the left STS [*t*(24) = −5.19, *P* < 0.001], which resulted in an overall rightward asymmetry of the STS volumes. On the subject level, the AI of the STS volumes ranged from −100.00 to 0.00 with 18 foetuses being right-lateralized and 7 foetuses being bilateral (AI = −20.00 to +20.00).

The STS depths and volumes in both the left and the right hemisphere were positively associated with the gestational age at the time of the foetal MRI examination (left STS_depth_: *r* = 0.87, *P* < 0.001; left STS_volume_: *r* = 0.88, *P* < 0.001; right STS_depth_: *r* = 0.91, *P* < 0.001; right STS_volume_: *r* = 0.86, *P* < 0.001), which suggests a bilateral effect of gestational age on STS depth and volume, and deeper sulci in older foetuses. The AI of the STS depths and volumes, however, did not correlate with the gestational age at scan (depth: *r* = 0.38, *P* = 0.060; volume: *r* = 0.21, *P* = 0.313), indicating that STS asymmetry did not significantly change across the examined gestational period. Moreover, no sex differences in the STS depths/volumes or AI of the STS depths/volumes were found (all *P* > 0.050).

### Neonatal speech discrimination

**Figure 1:**
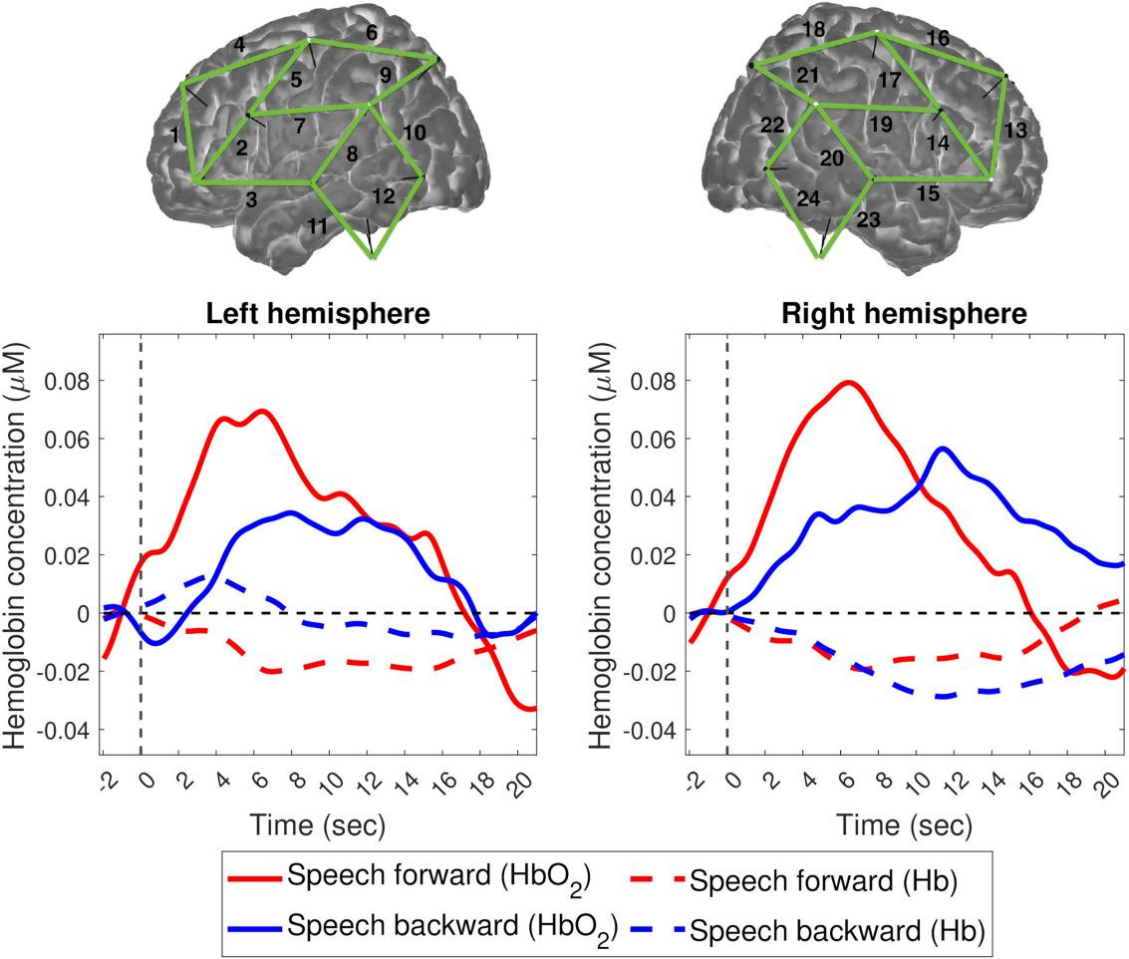
**Oxyhaemoglobin (HbO_2_) and deoxyhaemoglobin (Hb) concentration changes for forward and backward speech averaged across all subjects (*N* = 25) and all channels (in green) per hemisphere (*n* = 12).** Paired *t*-tests revealed no significant difference between conditions in the left [*t*(24) = 0.66, *P* = 0.517], i.e., channels 1 to 12, or the right hemisphere [*t*(24) = 0.33, *P* = 0.744], i.e., channels 13 to 24. Solid lines represent HbO_2_ concentration changes, while dotted lines represent Hb concentration changes. Red lines indicate forward speech, and blue lines indicate backward speech.

Across all fNIRS channels, paired *t*-tests did not reveal significant differences in neonates’ HbO_2_ mean concentration changes between speech conditions in the left or the right hemisphere (Table 2). However, visual inspection of the time courses of the two speech conditions does reveal a larger haemodynamic response to forward than backward speech in both hemispheres (Fig. 1).

**Table 2:** Oxyhemoglobin (HbO_2_) concentration during forward and backward speech, differences between speech conditions, and laterality indices

	Forward speech	Backward speech		
	Mean (SD), (Range)	Mean (SD), (Range)	Paired *t*-test	*P*-value
**All channels**				
Left hemisphere	0.04 (0.12), (−0.16–0.28)	0.02 (0.13), (−0.20–0.31)	0.66	0.517
Right hemisphere	0.05 (0.11), (−0.14–0.34)	0.03 (0.13), (−0.24–0.26)	0.33	0.744
**Activated channels**				
Left frontal cluster	0.06 (0.11), (−0.12–0.33)	0.01 (0.12), (−0.19–0.29)	1.94	0.064
Right temporal cluster	0.08 (0.13), (−0.13–0.39)	0.05 (0.13), (−0.19–0.19)	0.71	0.483
**Laterality index** for speech discrimination	−9.07 (40.04), (−99.87–56.86)			

The mean HbO_2_ concentration changes to both speech conditions varied widely between neonates, suggesting interindividual differences in the degree of neural activation during forward and backward speech (Table 2). While some neonates showed a strong haemodynamic response to speech in both hemispheres, some activated the left hemisphere more than the right, and some responded little to speech in either hemisphere. Importantly, as can be seen from the effect sizes reported in Supplementary Table 3, our single-subject analyses found larger variability in the degree to which neonates discriminated between speech conditions in the right hemisphere (*M*_effect size_ = −0.16, SD_effect size_ = 3.57) than in the left hemisphere (*M*_effect size_ = −0.12, SD_effect size_ = 2.87). It further revealed that five neonates exhibited significant speech discrimination only within the left hemisphere, three showed significant neural speech discrimination only in the right hemisphere, and six neurally discriminated in both hemispheres (Supplementary Table 3). Eleven neonates did not significantly discriminate speech from non-speech in either hemisphere.

The interindividual variability in HbO_2_ concentration changes to speech stimuli is also reflected in the wide range in laterality indices of speech discrimination (i.e., the difference between neural responses to forward and backward speech; Table 2, Supplementary Fig. 3). At the group level, the mean laterality index of neural speech discrimination across all channels showed a rightward shift. At the single-subject level, however, only 7 neonates exhibited rightward lateralization, while 10 showed bilateral activation (−0.20 to 0.20), and 8 displayed leftward lateralization of neural speech discrimination.

**Figure 2:**
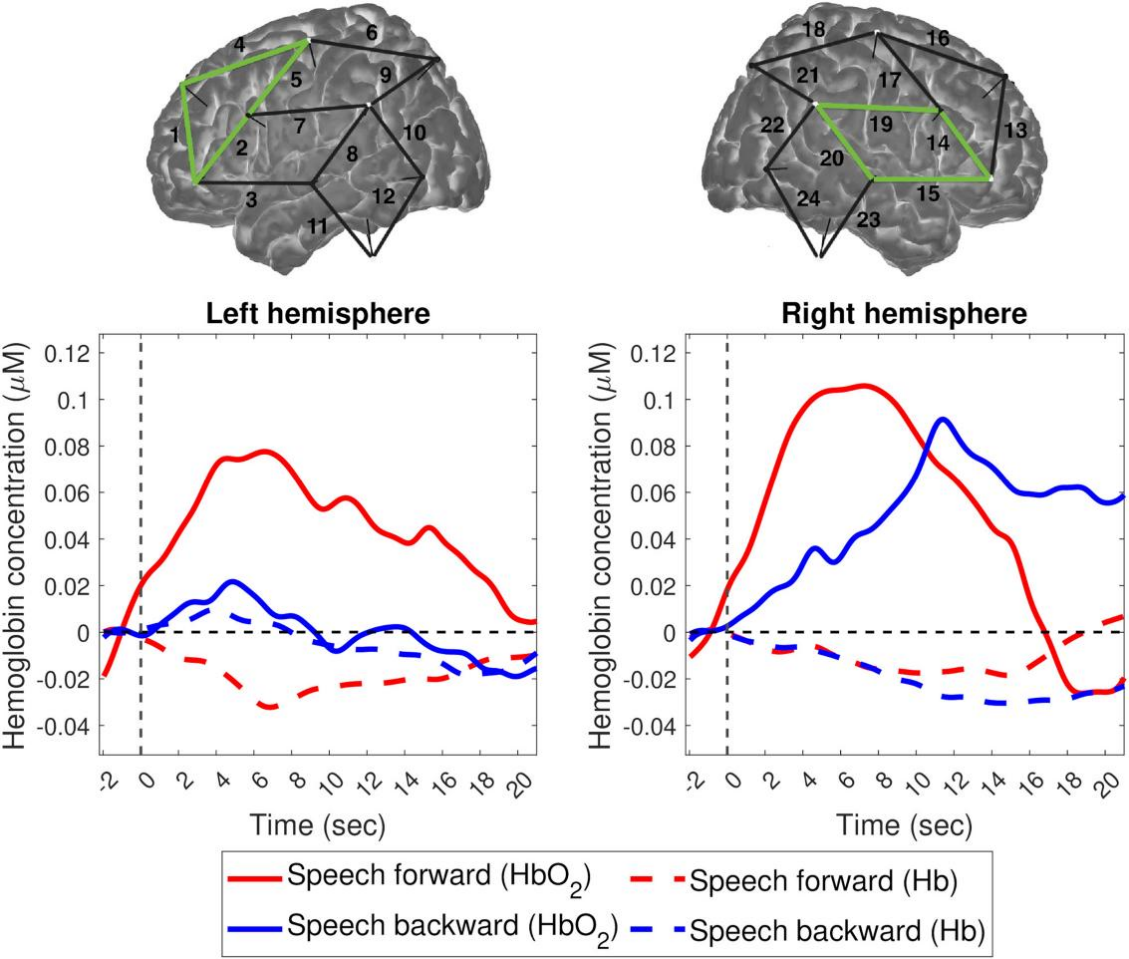
**Time course of oxyhaemoglobin (HbO_2_) and deoxyhaemoglobin (Hb) concentration changes for forward and backward speech in activated clusters (in green) in each hemisphere (*n* = 4), averaged across all subjects (*N* = 25).** Paired *t*-tests revealed a marginally significant difference between conditions in the left frontal cluster [*t*(24) = 1.94, *P* = 0.064], i.e., channels 1, 2, 4, and 5. No significant difference was found in the right temporal cluster [*t*(24) = 0.71, *P* = 0.483], i.e., channels 14, 15, 19, and 20. Solid lines represent HbO_2_ concentration changes, while dotted lines represent Hb concentration changes. Red lines indicate forward speech, and blue lines indicate backward speech.

With regard to the activated regions, the cluster-based permutation analysis revealed a left frontal and a right temporal cluster for forward speech and a right temporal cluster for backward speech (Fig. 2; see Supplementary Tables 4–7 for detailed results of the cluster-based permutation tests). Paired *t*-tests further revealed a marginally significant difference between speech conditions in the left frontal cluster (Table 2). As can be seen in the time course of the haemoglobin concentration changes, depicted in the bottom panels of Fig. 2, the left frontal region activated for forward, but not for backward speech, while the right temporal region showed haemodynamic changes to both forward and backward speech.

### Foetal structural brain asymmetry and neonatal speech discrimination

Foetal STS depth asymmetry negatively correlated with neural speech discrimination in the right hemisphere (*r* = −0.48, *P* = 0.014). This result remained stable when analyses were controlled for gestational age at scan (*r*_p_ = 0.42, *P* = 0.044). In contrast, STS depth asymmetry was not significantly associated with the functional lateralization of neural speech discrimination (Table 3, Supplementary Fig. 4, Supplementary Table 8).

**Table 3:** Correlational findings

	Foetal STS depth asymmetry	Foetal STS volume asymmetry
	*r* (*P*)	*r* (*P*)
**Mean HbO_2_ in the left hemisphere**		
Forward speech	−0.06 (0.790)	0.04 (0.863)
Backward speech	0.04 (0.843)	0.11 (0.586)
Difference between forward and backward speech	−0.07 (0.749)	−0.06 (0.784)
**Mean HbO_2_ in the right hemisphere**		
Forward speech	−0.58 (0.002)*	−0.38 (0.058)
Backward speech	0.22 (0.288)	0.10 (0.629)
Difference between forward and backward speech	**−0.48 (0.014)**	−0.29 (0.159)
**Laterality of neural speech discrimination**	0.15 (0.465)	0.21 (0.326)

Bold numbers indicate significance (*P* < 0.05). *Significance after Bonferroni correction (*P* < 0.0125) in the exploratory analyses of the individual speech conditions (forward/backward).

Exploratory analyses additionally revealed a significant association between foetal STS depth asymmetry and the mean HbO_2_ concentration change to forward speech in the right hemisphere (*r* = −0.58, *P* = 0.002; *r*_p_ = −0.52, *P* = 0.009). This indicates that subjects who showed less rightward asymmetry of the foetal STS depths at the time of their foetal MRI examination activated their right hemisphere less while listening to speech postnatally and discriminated less between speech and non-speech in their right hemisphere. In contrast, the AI of the STS depths did not correlate with any haemodynamic response in the left hemisphere, which included the mean HbO_2_ concentration change to forward speech, backward speech, and the difference between those two conditions. Furthermore, the asymmetry of the foetal STS volumes was not significantly associated with the neonatal haemodynamic response to forward speech, backward speech, or speech discrimination.

## Discussion

To the best of our knowledge, this is the first longitudinal, prospective study to examine the predictive value of STS asymmetry in neurologically healthy foetuses for language lateralization and neural speech discrimination shortly after birth. The majority of foetuses in this study exhibited a deeper and more voluminous STS in the right than the left hemisphere. Yet, the degree of asymmetry of the foetal STS depths and volumes varied between individuals. After birth, the overall group activated left frontal and right temporal regions during a speech discrimination paradigm. Again, there was variability in the degree of neural activation in response to speech. Most importantly, foetal STS depth asymmetry towards the left hemisphere was significantly associated with less right-hemispheric speech discrimination and with less neural activation in the right hemisphere while listening to speech shortly after birth.

### Variability in foetal STS asymmetry

Our first finding pertains to the STS asymmetry in the foetal brain. Similar to previous studies, we found a rightward asymmetry of the STS depths and volumes in the overall group, regardless of gestational age at the time of the foetal MRI examination.^5-7^ Moreover, the present study did not find sex differences in STS asymmetry, which further aligns with previous studies.^3,4^

The right STS, in addition to its greater depth, also develops 1–2 weeks earlier and matures faster than the left STS does, at around 23 weeks of gestation. Due to this early and asymmetric appearance of the STS during foetal brain development, a strong genetic influence has been suggested.^45^ Interestingly, recent studies have found that, despite an overall symmetric genetic control of sulcal pit formation across the brain, the STS has an asymmetric heritability, with a higher genetic influence in the left STS.^46,47^ As language is typically lateralized to the left hemisphere in the mature brain, a larger heritability within the left hemisphere would implicate stronger genetic constraints on the structural organization of the language network. Therefore, alterations in gene expression linked to language lateralization and asymmetric activation in the STS, such as FOXP2 and KIAA0319, respectively, could lead to interindividual variability in speech processing in the prenatal and early postnatal period.^48,49^ Taken together, even though the right STS appears earlier in the foetal brain, studies suggest variability in STS asymmetry to be driven by the left STS, which is known to be involved in language processing.^46,47^ Future language studies employing foetal MRI should thus consider directly analysing the left STS depth rather than depth asymmetry between hemispheres.

### Neonatal speech perception and discrimination

The cluster-based permutation test revealed an activated left frontal and a right temporal cluster for forward speech and a right temporal cluster for backward speech, with a marginally significant difference between conditions in the left frontal cluster. In contrast, group analyses across channels did not find significant differences between conditions in either hemisphere. However, at the individual level, our data revealed a wide range in neural activation in response to speech versus non-speech, with the majority of neonates showing significant neural speech discrimination in the left hemisphere or in both hemispheres, and only some discriminating only in the right hemisphere or little in either hemisphere. This finding contributes to a large body of evidence showing that neonates activate a left-lateralized language-specific network for forward but not for backward speech.^10,11,13,50^ It also reiterates the utility and value of single-subject analyses, particularly in developmental contexts. Similarly, the laterality indices of speech-specific activations (i.e., neural speech discrimination) across all channels ranged from left lateralization to bilateral activation and right lateralization. Given that language becomes increasingly lateralized towards the left hemisphere during childhood and adolescence,^51^ this early interindividual difference in the lateralization of neural speech discrimination may reflect different levels of maturity of the neonatal language network.

### Association between foetal STS asymmetry and neonatal speech discrimination

Our third and most important finding relates the anatomical asymmetry of the foetal STS to neonatal speech discrimination. Contrary to our initial hypothesis, foetal STS asymmetry did not significantly predict neonatal left-hemispheric speech discrimination. Instead, we found that foetal STS depth asymmetry predicted right-hemispheric neural speech discrimination and the neural response to forward speech in the right hemisphere of the neonatal brain. In other words, greater development of the left STS relative to its right counterpart was associated with reduced right-hemispheric, rather than increased left-hemispheric, involvement in both speech discrimination and speech processing.

Moreover, single-subject analyses revealed that there was less variability in the degree to which neonates discriminated in the left compared with the right hemisphere, which suggests that the left hemisphere may already be primed for speech-specific neural processing at birth. In contrast, right-hemispheric involvement appears to depend on the maturation of the individual language network. Consequently, foetal STS depth asymmetry also did not predict the lateralization of neural speech discrimination. This may be partly attributed to the lower variability in left-hemispheric neural speech discrimination, but it could also stem from the characteristics of the functional laterality index itself. As fNIRS data can have both positive and negative values, the absolute difference between conditions was employed to calculate the functional lateralization of neural speech processing, as previously applied in Bartha-Doering *et al*.^36^ However, it remains unclear whether using absolute values might result in a loss of information regarding haemodynamic responses. It could be hypothesized that negative responses also carry meaningful insights, although little is currently known about them.

In contrast to the significant findings regarding the predictive value of the foetal STS depth asymmetry on neonatal neural speech discrimination, we did not find the foetal STS volume asymmetry to be significantly associated with the neonatal haemodynamic response to forward or backward speech, nor with the difference between the two conditions. This underlines the findings of previous studies showing that foetal STS depth is a sensitive measure for structural brain asymmetry^5,35^ and later cognitive outcome,^30^ while, to the best of our knowledge, no study so far has reported foetal STS volume to be of similar predictive value. In addition, we found no correlation between foetal STS asymmetry and mean Hb concentration changes to forward or backward speech in either hemisphere, which further underlines the idea that HbO_2_ is a stronger and more consistent measure of cortical activation, particularly in infants.^41,42^

Structural asymmetries in language-related regions appear early in foetal brain development, with studies reporting a longer left temporal lobe and a deeper right STS by 23 weeks of gestation.^5^ About 2 weeks later, around 25 weeks of gestation, the auditory network becomes functional, and foetuses start experiencing different types of sounds, including the mother’s heartbeat, movement, and voice.^52^ Around the same time, the left STS appears.^5^ In older children and adults, auditory perception is processed in the STS bilaterally, whereas most of the language-specific processing occurs in the left STS.^53^ This activation pattern has already been demonstrated in foetuses during the third trimester of pregnancy, with one study finding significant activation in response to auditory stimuli in the left auditory cortex at 33 gestational weeks.^54^ In turn, the presently observed relationship between foetal STS depth asymmetry and neonatal speech discrimination likely reflects a developmental continuity between early structural and later functional brain organization. While a rightward depth asymmetry of the STS is characteristic of the human brain, interindividual variability in the degree and direction of this asymmetry may signal subtle differences in the timing and trajectory of hemispheric maturation. Such structural variations could, in turn, influence the establishment of functional lateralization in the developing language network. Given this, our findings suggest that prenatal STS depth asymmetry does not represent an isolated anatomical feature but rather an early marker of hemispheric specialization, shaping how the brain begins to process speech stimuli.

Several studies have similarly found an anatomo-functional correspondence between the STS structure and language function in healthy children and adults.^30,46,55^ Two of these studies demonstrated that children with STS depth asymmetry towards the left hemisphere had better verbal abilities, compared with children with rightward asymmetry, who tended to have average or below average verbal abilities.^30,55^ Moreover, Bartha-Doering *et al.*^30^ found that the depth asymmetry of the STS at the foetal stage correlated with language lateralization 6–13 years later, with children showing asymmetry towards the left hemisphere also demonstrating increased left language localization. Finally, one study in healthy adults from the Human Connectome Project found STS depth asymmetry towards the left hemisphere to correlate with increased left-hemispheric activations during a language task, with a deeper left STS driving this association.^46^ While these studies demonstrated a relationship between STS depth asymmetry and language function in older children and adults, the current study is the first to show an association between foetal STS depth asymmetry and neural speech discrimination in neurologically healthy neonates.

However, the association between STS asymmetry and language function continues to be debated, particularly in adults. For example, Specht and Wigglesworth^56^ found no relationship between anatomical asymmetries of the STS and brain activations during a speech perception task. One reason for this difference in findings may be that, unlike foetal STS depth asymmetry, the anatomical asymmetry in adults is associated with a higher occurrence of ‘plis de passage’ in the left than in the right STS.^4,53^ Since these sulcal interruptions can influence the accuracy of the STS depth measurements, the foetal period may provide a unique window of opportunity during which STS depth asymmetry is predictive of later language abilities. Moreover, one recent study found that the functional, but not the structural asymmetry of the STG in the infant brain predicted children’s language abilities 4–5 years later.^15^ While their functional findings align with our results, our anatomical data are not entirely comparable, as their study examined the volume, cortical thickness, and surface area of the STG whereas we measured the depth and volume of the STS. The STS depth, particularly at the foetal stage, may thus be a more sensitive predictor of later language abilities than the STG volume.

### Limitations and future directions

The current study has several limitations that should be acknowledged. First, due to its prospective, longitudinal design, participant recruitment was slow, resulting in a relatively small sample size of 35 foetuses. Moreover, collecting infant fNIRS data can be challenging, as excessive head motion often introduces noise. Even though the preprocessing stream should correct these motion artefacts, data can still become unusable when too many channels or trials are removed. This led to the exclusion of 10 infants in the present analysis, further reducing the sample size. Second, while all infants included in the final analysis were sleeping or resting with their eyes closed, their sleep states were not recorded during data acquisition. Thus, differences in sleep state may have contributed to variability in neonatal brain activation patterns. It will be important for future research to monitor sleep state simultaneously using electroencephalography (EEG) when examining neural activity in neonates and young infants. Finally, four foetuses in the current sample did not have a visible STS in either hemisphere, despite meeting the gestational age inclusion criteria. It remains unclear whether these foetuses exhibit delayed gyrification, which might be clinically relevant and could also lead to delayed language processing, or whether they simply reflect normal variability in the developmental trajectory of STS formation. Future studies will be required to determine whether the absence of a visible STS in some foetuses represents delayed cortical folding with potential implications for language development, or whether it falls within the range of normal developmental variability. In light of these limitations, the present findings should be interpreted with caution and replicated in a larger cohort of neurologically healthy foetuses. However, given the lack of studies on the association between foetal STS asymmetry and neonatal speech discrimination, these results provide valuable first insights into the potential anatomo-functional correspondence between the STS structure and language function in neurologically healthy children.

## Supplementary Material

fcag048_Supplementary_Data

## Data Availability

The data that support the findings of this study are available from the corresponding author, upon reasonable request.
