# Supplementary material for: Predictive value of foetal superior temporal sulcus asymmetry for neonatal speech discrimination
Source: Brain Commun. 2026 Feb 13;8(1):fcag048. doi: 10.1093/braincomms/fcag048 (PMC12946156; doi:10.1093/braincomms/fcag048)
Supplement: fcag048_Supplementary_Data [file fcag048_supplementary_data.pdf]

Supplementary material

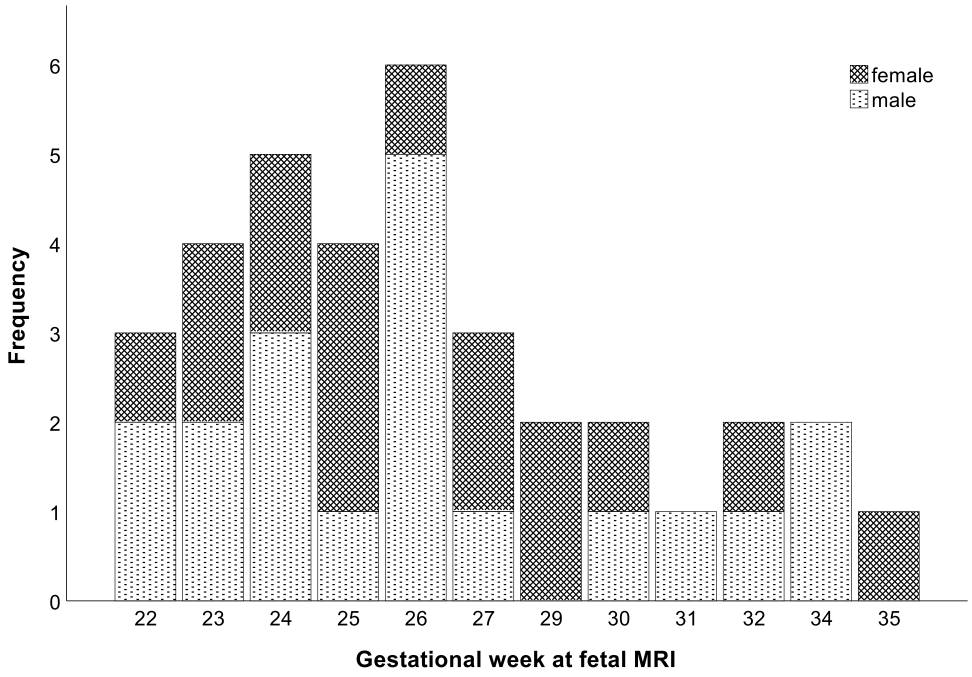

Supplementary Fig. 1. Distribution of gestational ages (in weeks) at the time of the fetal MRI examination.

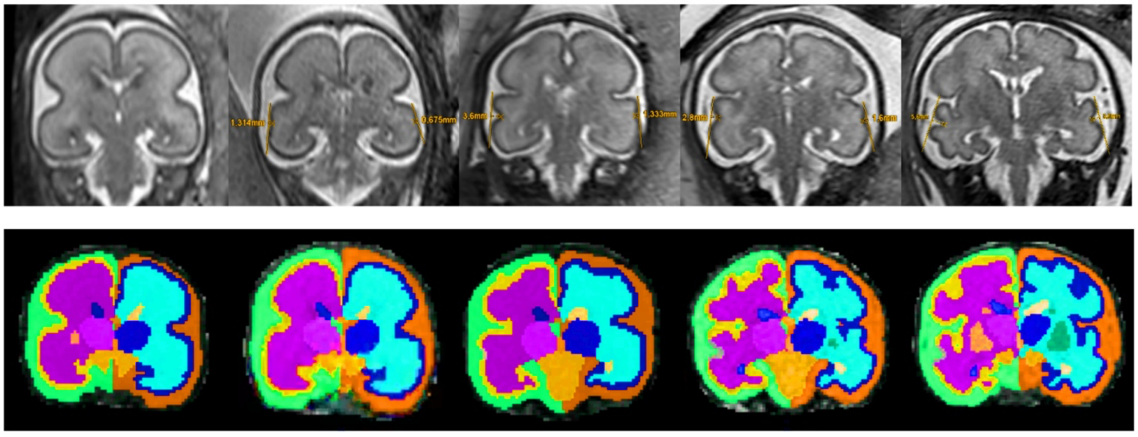

Supplementary Fig. 2. Top: Coronal T_2-weighted images of fetal brains at 23, 25, 27, 29, and 31 gestational weeks, including STS depth measurements. Bottom: Coronal view of super-resolution images of fetal brains at 23, 25, 27, 29, and 31 gestational weeks with BOUNTI automatic segmentations and manual STS segmentations. The figure design was inspired by Kasprian *et al.*[1]

Supplementary Note 1.

Deoxyhemoglobin (Hb) results. Paired t-tests did not find a significant difference in Hb concentration changes between forward and backward speech in the left ($t(24) = -1.45$, $P = .159$), or in the right hemisphere ($t(24) = .41$, $P = .685$). Pearson's correlations also did not reveal a significant association between the asymmetry of the fetal STS depths/volumes and Hb concentration changes to forward or backward speech in either hemisphere (all $P > .05$).

Supplementary Table 1. Fetal brain volumes of fetuses (n = 25).

	Fetal Brain Volume (cm^3)		
	**Mean**	**SD**	**Range**
**Cortical grey matter**	34.61	20.14	14.58 – 89.86
**White matter**	73.01	31.31	33.48 – 146.84
**Cavum**	.51	.33	.28 – 1.74
**Brainstem**	3.07	1.11	1.71 – 5.48
**Cerebellum**	5.38	3.35	2.10 – 14.10
**Deep grey matter**	6.90	2.94	3.20 – 12.85
**STS**	.46	.50	.00 – 1.85
**Total brain volume**	123.49	58.56	55.47 – 270.86

Supplementary Table 2. Left-right hemispheric differences in fetal brain volumes.

	**Left Hemisphere**	**Right Hemisphere**	**Asymmetry Index**	
	**Mean (SD), [Range]**	**Mean (SD), [Range]**	**Mean (SD), [Range]**	**Paired t-test (*P*)**
**Cortical grey matter (cm^3)**	17.33 (10.08), [7.29, 44.91]	17.28 (10.07), [7.29, 44.95]	.19 (1.25), [-1.59, 2.88]	.58 (.569)
**White matter (cm^3)**	36.38 (15.49), [16.80, 73.18]	36.64 (15.82), [16.78, 73.66]	-.26 (.79), [-2.15, 1.07]	-1.99 (.058)
**Deep grey matter (cm^3)**	3.44 (1.47), [1.60, 6.38]	3.45 (1.47), [1.60, 6.47]	-.15 (1.12), [-2.42, 2,65]	-.55 (.587)
**Superior temporal sulcus (cm^3)**	.14 (.21), [.00, .59]	.32 (.31), [.00, 1.27]	-48.59 (42.40), [-100.00, .00]	**-5.19 (<.001)**

Supplementary Table 3. Speech discrimination results per subject.

Subject Nr.	Left Hemisphere t (*P*)	Right Hemisphere t (*P*)
1	2.16 (.056)	-1.53 (.170)
2	**4.27 (.001)**	1.55 (.150)
3	1.80 (.100)	-1.27 (.239)
4	.63 (.549)	2.08 (.064)
5	-.18 (.858)	.93 (.374)
6	**-6.79 (<.001)**	**-4.07 (.002)**
7	**-3.35 (.009)**	-.49 (.637)
8	**4.73 (<.001)**	**8.81 (<.001)**
9	-.78 (.464)	**3.13 (.014)**
10	.55 (.599)	-1.08 (.318)
11	**2.35 (.041)**	**4.00 (.002)**
12	-.26 (.803)	-.18 (.861)
13	**-5.39 (<.001)**	**-10.52 (<.001)**
14	1.35 (.212)	1.28 (.232)
15	**2.29 (.045)**	-1.34 (.251)
16	.00 (.999)	**-2.77 (.024)**
17	1.56 (.150)	1.65 (.134)
18	**2.30 (.047)**	**3.88 (.003)**
19	-2.18 (.052)	**-2.53 (.028)**
20	-1.33 (.212)	.68 (.515)
21	**2.79 (.039)**	1.95 (.087)
22	-1.78 (.119)	-1.14 (.284)
23	**-3.85 (.003)**	**-4.18 (.002)**
24	**-3.35 (.009)**	-1.64 (.129)
25	-0.47 (.650)	-1.17 (.279)

Bold numbers indicate significance ($P < 0.05$).

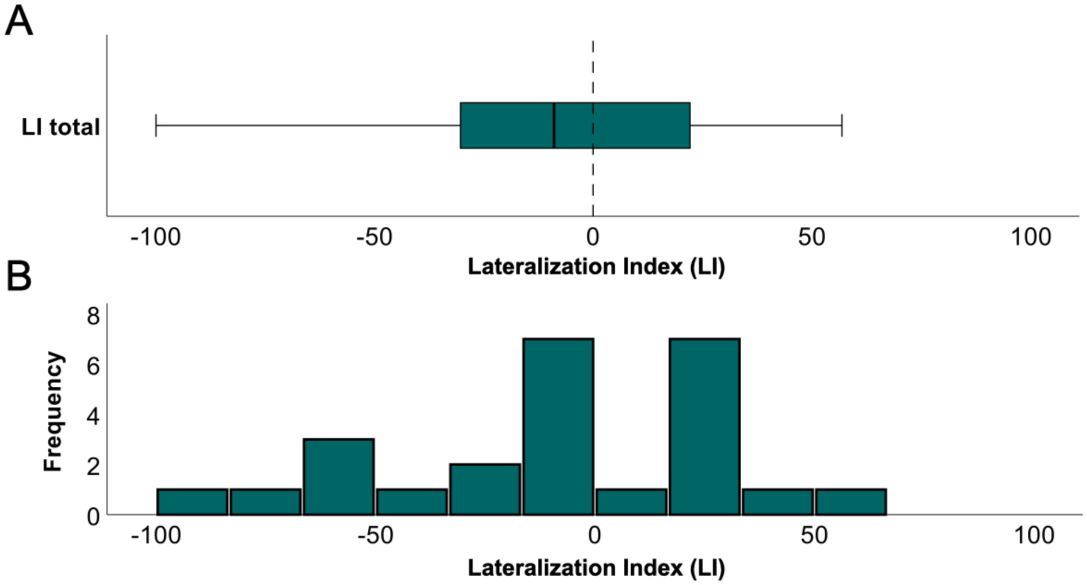

Supplementary Fig. 3. (A) Lateralization index (LI) of neural speech discrimination abilities in all neonates (n = 25) across all channels (LI total). The LI is determined by ((|Left| – |Right|)/(|Left| + |Right|))*100, with -100 indicating complete right functional lateralization and +100 indicating complete left functional lateralization. (B) Frequency distribution of the LIs of all 25 subjects.

Supplementary Table 4. Permutation test for each individual channel for forward speech versus the zero baseline.

Channel	Multiple time sample-specific t	df	Permutation *P*
1	90.85	24	.109
**2**	**228.67**	**24**	**.029**
3	0.00	20	1.000
4	130.47	22	.087
5	56.89	23	.173
6	0.00	17	1.000
7	15.67	21	.245
8	24.60	21	.228
9	-33.87	22	.218
10	23.01	22	.246
11	129.50	16	.099
12	21.41	19	.276
13	0.00	23	1.000
**14**	**283.85**	**22**	**.022**
**15**	**207.11**	**22**	**.016**
16	0.00	23	1.000
17	0.00	23	1.000
18	0.00	18	1.000
**19**	**266.31**	**21**	**.023**
20	0.00	20	1.000
**21**	**268.10**	**18**	**.008**
**22**	**227.09**	**20**	**.027**
23	9.03	14	.353
24	0.00	17	1.000

Bold numbers indicate significance ($P < 0.05$). A multiple time sample-specific t-value of 0 signifies that the channel did not surpass the predefined threshold of ±2.

Supplementary Table 5. Cluster-based permutation test results for forward speech.

Channels in cluster	Forward HbO_2 vs. zero baseline	
	Multiple sample-specific t	Permutation *P*
**1, 2, 4, 5**	**506.89**	**.034**
2, 3, 7, 8	268.94	.125
5, 6, 7, 9	38.69	.529
8, 10, 11, 12	198.52	.181
13, 14, 16, 17	283.85	.125
**14, 15, 19, 20**	**757.27**	**.008**
**17, 18, 19, 21**	**534.41**	**.022**
20, 22, 23, 24	236.12	.122

Bold numbers indicate significance ($P < 0.05$).

Supplementary Table 6. Permutation test for each individual channel for backward speech versus the zero baseline.

Channel	Multiple time sample-specific t	df	Permutation *P*
1	0.00	24	1.000
2	37.02	24	.195
**3**	**211.53**	**20**	**.035**
4	0.00	22	1.000
5	0.00	23	1.000
6	0.00	17	1.000
7	0.00	21	1.000
8	0.00	21	1.000
9	0.00	22	1.000
10	0.00	22	1.000
11	0.00	16	1.000
12	-20.13	19	.194
13	0.00	23	1.000
14	0.00	22	1.000
**15**	**332.82**	**22**	**.013**
16	0.00	23	1.000
17	0.00	23	1.000
18	0.00	18	1.000
19	0.00	21	1.000
**20**	**195.56**	**20**	**.035**
21	0.00	18	1.000
22	0.00	20	1.000
23	0.00	14	1.000
24	0.00	17	1.000

Bold numbers indicate significance ($P < 0.05$). A multiple time sample-specific t-value of 0 signifies that the channel did not surpass the predefined threshold of ±2.

Supplementary Table 7. Cluster-based permutation test results for backward speech.

Channels in cluster	Backward HbO_2 vs. zero baseline	
	Multiple sample-specific t	Permutation *P*
1, 2, 4, 5	37.02	.516
2, 3, 7, 8	248.55	.158
5, 6, 7, 9	0.00	1.000
8, 10, 11, 12	-20.13	.590
13, 14, 16, 17	0.00	1.000
**14, 15, 19, 20**	**528.38**	**.029**
17, 18, 19, 21	0.00	1.000
20, 22, 23, 24	195.56	.185

Bold numbers indicate significance ($P < 0.05$). A multiple time sample-specific t-value of 0 signifies that none of the channels in the cluster candidate surpassed the predefined threshold of ±2.

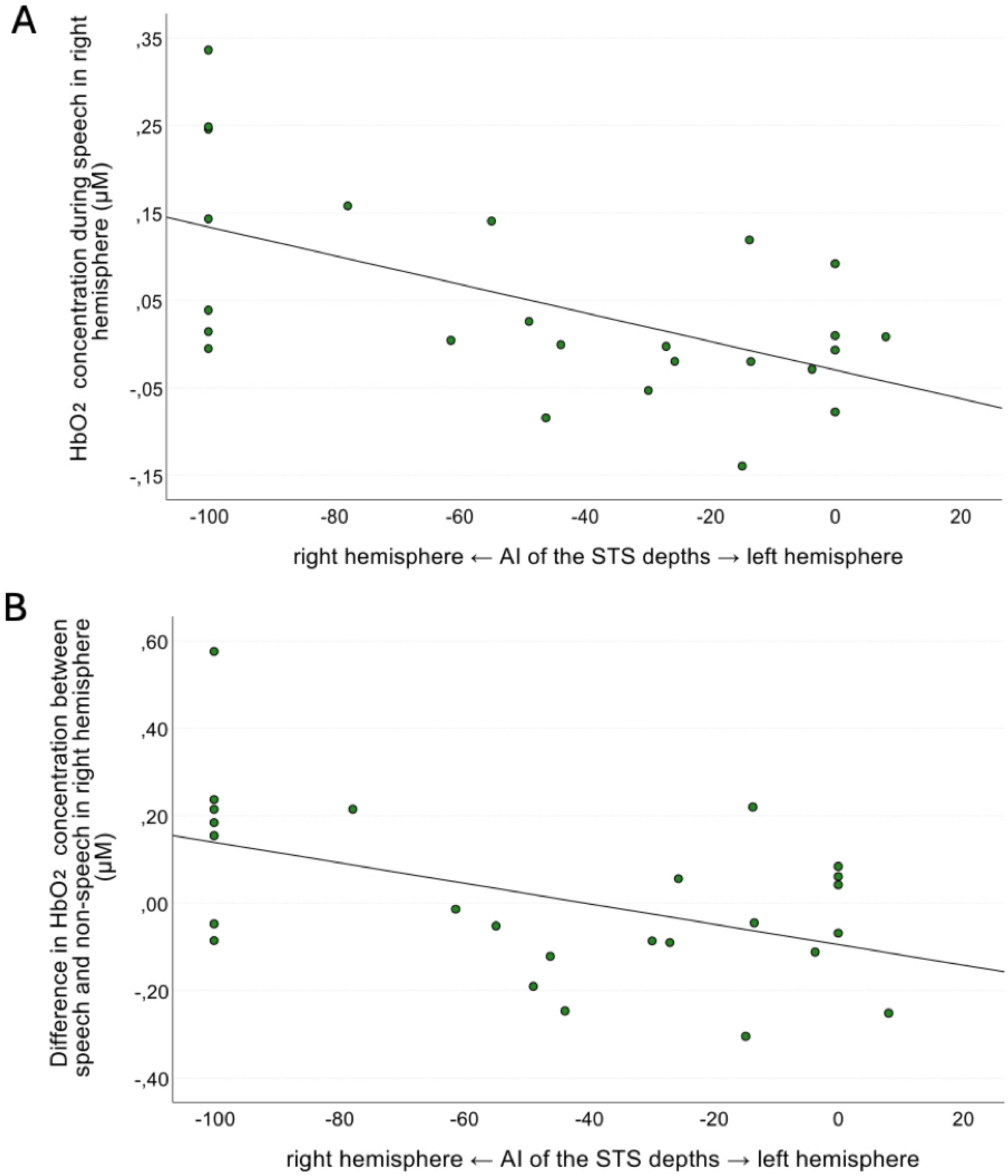

Supplementary Fig. 4. (A) Pearson correlation between the asymmetry index (AI) of the STS depths and the HbO_2 concentration change during forward speech in the right hemisphere (r = -.58, p = .002). (B) Pearson correlation between the AI of the STS depths and the difference in HbO_2 concentration change between forward and backward speech in the right hemisphere (r = -.48, p = .014). Each data point represents one subject's STS depth asymmetry and corresponding HbO_2 concentration change during forward speech (A) or the difference between conditions (B).

Supplementary Table 8. Correlational results controlled for gestational age at fetal MRI.

	**Fetal STS depth asymmetry** r_P **(*P*)**	**Fetal STS volume asymmetry** r_P **(*P*)**
**Mean HbO_2 in the left hemisphere**		
Forward speech	-.07 (.736)	.03 (.886)
Backward speech	.13 (.558)	.16 (.452)
Difference between forward and backward speech	-.14 (.518)	-.09 (.663)
**Mean HbO_2 in the right hemisphere**		
Forward speech	**-.52 (.009)***	-.34 (.102)
Backward speech	.16 (.451)	.06 (.770)
Difference between forward and backward speech	**-.42 (.044)**	-.24 (.255)
**Laterality of neural speech discrimination**	.16 (.443)	.21 (.328)

Bold numbers indicate significance ($P < .05$). * indicates significance after Bonferroni correction ($P < .0125$) in the exploratory analyses of the individual speech conditions (forward/backward).

References

1. Kasprian G, Langs G, Brugger PC, et al. The prenatal origin of hemispheric asymmetry: an in utero neuroimaging study. *Cereb Cortex*. May 2011;21(5):1076-83. doi:10.1093/cercor/bhq179
